# Dysregulation of Anti-Inflammatory Annexin A1 Expression in Progressive Crohns Disease

**DOI:** 10.1371/journal.pone.0076969

**Published:** 2013-10-10

**Authors:** Angela Sena, Irina Grishina, Anne Thai, Larissa Goulart, Monica Macal, Anne Fenton, Jay Li, Thomas Prindiville, Sonia Maria Oliani, Satya Dandekar, Luiz Goulart, Sumathi Sankaran-Walters

**Affiliations:** 1 Department of Medical Microbiology and Immunology, University of California Davis, Davis, California, United States of America; 2 Nanobiotechnology Laboratory, Institute of Genetics and Biochemistry, Federal University of Uberlandia, Uberlandia, Minas Gerais, Brazil; 3 UCDHS: Division of Hepatology and Gastroenterology, University of California Davis, Davis, California, United States of America; 4 Department of Biology, Sao Paulo State University, UNESP, Sao José do Rio Preto, SP, Brazil; Institut Pasteur de Lille, France

## Abstract

**Background:**

Development of inflammatory bowel disease (IBD) involves the interplay of environmental and genetic factors with the host immune system. Mechanisms contributing to immune dysregulation in IBD are not fully defined. Development of novel therapeutic strategies is focused on controlling aberrant immune response in IBD. Current IBD therapy utilizes a combination of immunomodulators and biologics to suppress pro-inflammatory effectors of IBD. However, the role of immunomodulatory factors such as annexin A1 (ANXA1) is not well understood. The goal of this study was to examine the association between ANXA1 and IBD, and the effects of anti-TNF-α, Infliximab (IFX), therapy on ANXA1 expression.

**Methods:**

ANXA1 and TNF-α transcript levels in PBMC were measured by RT PCR. Clinical follow up included the administration of serial ibdQs. ANXA1 expression in the gut mucosa was measured by IHC. Plasma ANXA1 levels were measured by ELISA.

**Results:**

We found that the reduction in ANXA1 protein levels in plasma coincided with a decrease in the ANXA1 mRNA expression in peripheral blood of IBD patients. ANXA1 expression is upregulated during IFX therapy in patients with a successful intervention but not in clinical non-responders. The IFX therapy also modified the cellular immune activation in the peripheral blood of IBD patients. Decreased expression of ANXA1 was detected in the colonic mucosa of IBD patients with incomplete resolution of inflammation during continuous therapy, which correlated with increased levels of TNF-α transcripts. Gut mucosal epithelial barrier disruption was evident by increased plasma bacterial 16S levels.

**Conclusion:**

Loss of ANXA1 expression may support inflammation during IBD and can serve as a biomarker of disease progression. Changes in ANXA1 levels may be predictive of therapeutic efficacy.

## Introduction

Inflammatory bowel disease (IBD) is a debilitating disorder characterized by severe inflammation of the gastrointestinal tract, often leading to physical symptoms of abdominal pain and recurrent diarrhea [1,2]. Ulcerative Colitis (UC) and Crohns Disease (CD) are the two most common forms of IBD. The course of IBD varies among patients and includes a wide spectrum of complications such as intestinal hemorrhage, toxic megacolon, abscess and stricture formation, and fistulizing disease. One of the mechanisms of IBD includes the breakdown of gut homeostasis that may be induced by dysfunction in mucosal immunity [2-4]. Both human and murine studies suggest that several genetic defects in innate immunity and aberrant T-cell activation play a critical role in the pathogenesis of IBD [5,6]. Additionally, studies using an *in-vivo* murine model suggest that IBD symptoms may be attributed to TNF-α-induced intestinal T-cell activation [7]. In the last decade, administration of anti-TNF-α antibodies (Infliximab, IFX) has been effective in treating subsets of IBD patients. IFX therapy was shown to produce early changes in the gene expression profiles of intestinal epithelial cells that were predictive of clinical response [8]. 

Several investigations have focused on the identification of biomarkers of IBD progression that could be valuable in the diagnosis and treatment of IBD [9]. Availability of predictive correlates of clinical response would enable clinicians to determine the benefits or risks of initiating biologic therapy on an individual basis [8,10,11]. The majority of IBD biomarkers are correlates of inflammation. However, limited information is available on the anti-inflammatory processes and biomarkers in IBD and whether dysfunction in anti-inflammatory pathways contributes to the progression of IBD. Annexin A1, an anti-inflammatory factor, is a 37kDa calcium-dependent phospholipid binding protein, originally reported as glucocorticoid-induced protein with anti-phospholipase activity [12-14] that has been shown to regulate diverse cellular functions in several cell types. ANXA1 also exhibits profound inhibitory actions on leukocyte transmigration and activation, leading to the resolution of inflammation [15-18]. Its protective and anti-inflammatory role has been demonstrated in the animal models of endotoxemia, peritonitis, arthritis, and cerebral and myocardial ischemia [19-26]. Additionally, it is implicated in wound healing, especially in the setting of intestinal inflammation and injury [27,28]. It also has been shown to promote healing of indomethacin-induced gastric ulcers [28] and prevent intestinal mucosal injury in the murine model [29]. Previous studies reported conflicting findings about the ANXA1 expression in IBD. ANXA1 expression is decreased in the subcellular fraction of intestinal epithelial cells from patients with ulcerative colitis as compared to healthy controls, while other studies found an increase in ANXA1 expression [28,30]. Thus the role of ANXA1 in IBD and its relationship to systemic inflammation is unclear. 

In this study, we investigated the role of ANXA1 associated anti-inflammatory processes in the development of IBD and during IFX therapy. We measured ANXA1 expression in peripheral blood and gut biopsy samples of patients with CD on IFX therapy. The complete loss of ANXA1 protein was detected in colonic tissues from chronic CD patients which correlated with clinical status, response to therapy, TNF-α expression, and lymphocyte activation. Our findings suggest that loss of ANXA1-mediated anti-inflammatory function may be a potential mechanism of immune dysfunction in the development of IBD. 

## Materials and Methods

### Study Participants

Study participants were enrolled at the University of California, Davis, Medical Center, in Sacramento, California. IBD patients with a previous diagnosis of Crohns Disease (CD) (n = 28) and healthy controls with no prior history of IBD (n = 12) were enrolled in the study (Table 1). The study group and control group were matched for ethnicity with > 90% of participants being white/Caucasian. The CD patient group (consisting of 16 participants on long term longitudinal follow-up of treatment long term IFX and 12 participants with a single time point following therapy) was identified based on patients’ clinical history and an “standard of care” endoscopic evaluation. Longitudinal peripheral blood samples (20ml) were obtained from all participants. Perioperative gut resection (n=2, from IBD patients who were unresponsive to medical management with immunosuppressive agents) or colonic mucosal biopsy samples (n=6) from selected IBD participants were obtained. Gut biopsy samples from IBD patients were obtained from the disease affected region of colon and from an adjacent normal/unaffected area of the colon of same patients. Colonic biopsies were also obtained from 5 healthy volunteer participants. Ileo-colic biopsies of healthy volunteers and IBD patients were obtained by colonoscopy. IBD patients were provided therapy based on current therapeutic recommendations. All participants completed an IBD questionnaire (IBDQ) at every visit. The IBDQ is a validated and standardized health related quality of life questionnaire commonly used to assess response to IBD treatment. Scores range from 34-224 with a higher score indicating a better quality of life. The institutional review board at the University of California, Davis, approved this study protocol. Written consent was obtained from all participants as per IRB protocol.

**Table 1:** Patient characteristics.

	**Groups**	
	**IBD n (%)**	**NC n (%)**
**Total subjects**	28	12
**Sex**		
Male	13 (46%)	6 (50%)
Female	15 (54%)	6 (50%)
**Age**	41 (21-70)	35 (27-44)
**Medications**		
Infliximab	24(88%)	N/A
AZA/6-MP/6-TG	19 (67%)	N/A
Methotrexate	3 (9%)	N/A
Prograf	5 (15%)	N/A
Steroids	9 (30%)	N/A
Mesalamines	11 (39%)	N/A

Note: n = total subjects. Within parentheses: percentage of patients. Abbreviations: IBD (inflammatory bowel disease), NC (normal controls), N/A (not applicable).

### Real-Time PCR

DNA and RNA were extracted from isolated PBMC and plasma samples (Qiagen DNeasy and RNeasy extraction kits, Qiagen, Valencia) [31,32]. Real-time PCR assay (Taqman) was used to determine the mRNA levels of ANXA1 and TNF-α in PBMC. Primer-probe pairs for ANXA1 (Hs00167549_m1), TNF-α (Hs01113624_g1) and 16S rDNA (Panbakt 923f1: AACTCAAAGGAATTGACGGGG, Panbakt 923f2: AACTCAAATGAATTGACGGGG, Panbakt 1124r: GCTCGTTGCGGGACTTA, Panbakt 1075P: TGTCGTCAGCTCGTG ) (Applied Biosystems, CA) were tested and validated to have an amplification efficiency of >95%, comparable to that of glyceraldehyde-3-phosphate dehydrogenase (GAPDH). Relative mRNA (ANXA1 and TNF-α) expression levels were calculated from normalized Δ*C*_*T*_ (cycle threshold) values and are reported as the fold-change in expression. *C*_*T*_ values correspond to the cycle number at which the fluorescence signal exceeds the background fluorescence (threshold). In this analysis, the *C*_*T*_ value for the housekeeping gene (GAPDH) was subtracted from the *C*_*T*_ value of the target gene for each sample for normalization. For the detection of changes in gene expression in participant groups the RNA levels for each gene were compared with the levels in study group and are presented as the change in expression of each gene (ΔΔ*C*_*T*_). The values were converted to a linear scale (2^ΔΔCT^) (user bulletin 2; ABI Prism 7900 Sequence Detection System; Applied Biosystems, CA) [33,34]. Based on a previously determined standard curve, universal bacterial 16S rDNA levels in plasma samples were analyzed by real-time PCR assay using an ABI Prism 7900 sequence detector (Applied Biosystems, CA) [11]. Detection of the 16S rDNA product in plasma samples by real time PCR assay was confirmed by DNA sequencing.

### ELISA

The Annexin A1 (USCN Life Sciences Inc, Houston, TX) and CRP (Invitrogen, Camarillo, CA) levels in plasma samples were determined by ELISA. Briefly, Plasma samples were diluted to 1:150 (for ANXA1) or 1:3000 (for CRP) and used on a precoated ELISA plate as per manufacturer’s suggestions. Absorbance was measured using an SCA, xMark Absorbance Reader (Bio-Rad, Hercules, CA) and sample readings were extrapolated against a concurrently run standard curve. The values were output as Annexin A1 pg/ml of plasma and CRP mg/l of plasma. 

### Immunohistochemistry

Colonic tissue biopsy sections were obtained from healthy controls (n=5), IBD patients who were responsive to infliximab (n=6) and from colonic resections of IBD patients who were not responsive to infliximab therapy and required partial resection surgery (n=2). Gut mucosal samples were stored in CryoPrep (American Master Tech Scientific, Lodi). Immunohistochemical analysis was performed by incubating tissue sections overnight with a 1:100 dilution of mouse anti–ANXA1 (AbCam, San Francisco, CA) and reacting with a 1:100 dilution of rabbit FITC-conjugated anti-mouse (BioGenex, San Ramon, CA). DAPI was utilized to visualize nuclei [31,33]. Negative controls consisted of tissue sections reacted with no primary antibody and non-specific antibody containing serum. Images were captured by confocal laser microscopy using LSM 5 and PASCAL software (Zeiss, New York) and processed using the ZEN 2009 software.

### Immunophenotypic Analysis

Peripheral blood mononuclear cells (PBMC) were isolated by Ficoll gradient   and stained with Live/Dead Fixable Dead Cell Stain (Life Technologies, San Jose, CA). PBMC were stained with antibodies to CD3 (clone: UCHT1, Biolegend, San Diego, CA), CD4 (clone: OKT4, ebioscience, San Diego, CA), CD8 (clone: RPA-T8, Biolegend), CD19 (clone: H1B19, Biolegend), CD38 (clone: HIT-2, BD Bioscience, San Jose, CA), and CD45RO (clone: UCHL1, Life Technologies) followed by fixation with 1% Paraformaldehyde (Sigma, St. Louis, MO). Samples were collected using a modified LSRII flow cytometer (BD Bioscience) with a minimum of 500,000 events collected as previously published . Live lymphocytes were identified and gated by size, granularity and negative staining for the viability dye. B and T cell populations were further analyzed by specific staining.  Immunophenotypic analysis was performed using FlowJo v.8.5.2 (Treestar, Ashland, OR) [31,32,35].  

### Statistical Analysis

The data on cell activation, ANXA1, TNF-α and 16S were analyzed using ANOVA, unpaired t-test (two-tailed), Mann-Whitney, and their correlations with other variables were performed with linear regression. Annexin A1 levels in plasma were analyzed using unpaired and paired T test as necessary. P values <.05 is designated by a *. Statistical software included GraphPad Prism version 5.00 for Windows (GraphPad Software, San Diego). 

## Results

### Characteristics of Study Participants

The study population consisted of approximately 46% males in the IBD group and 50% in the control groups (Table 1). Study participants were matched for age, sex, and ethnicity. The average age of the control participants was 35 years while that of the IBD group was 41 years of age. The ethnic distribution among the patient groups was comparable with greater than 90% of participants being white/causasian. In the IBD group, all 28 patients were diagnosed with Crohn’s Disease (CD) by colonoscopy. Diagnosis of CD was made from histopathological analysis by the clinical laboratory services [11]. Most of the IBD patients had ileo-colic disease with minimal flares. A majority of the patients were either on or initiating Infliximab (IFX) therapy (88%) or AZA/6MP/6TG (67%) therapy or both (64%). Four IBD patients chose to not initiate therapy while 2 patients who were unresponsive to therapy underwent partial resection as a surgical intervention. 

### IFX therapy modifies T cell activation in IBD patients

**Figure 1:**
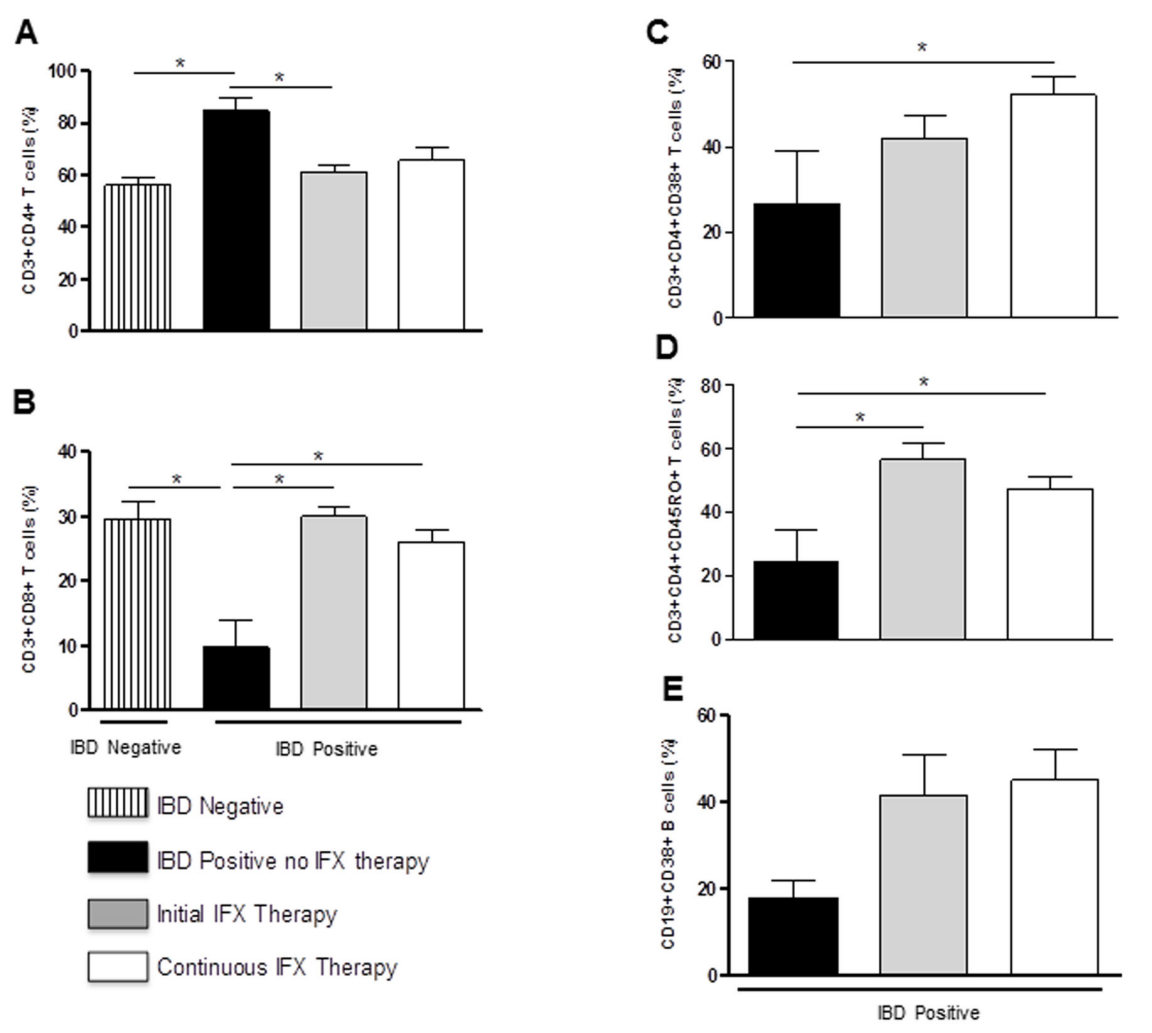
Effect of IFX on lymphocyte counts and cell activation. Flow cytometric analysis of peripheral blood samples from IBD patients with or without IFX therapy. A-E summarize the immunophenotypes (CD4+, CD8+, CD4+CD38+, CD4+CD45RO+, CD19+CD38+, respectively). Values are expressed as mean ± SEM of percentage of cell percentages. (A) A significant increase in IBD patients in CD4+ T cells compared to IBD negative healthy controls with a significant decrease in patients initiating IFX therapy was observed. (B) A significant increase in CD8+ T cells was observed in patients initiating IFX therapy as well as those on continuous IFX therapy compared to non-treated IBD patients. Patients on continuous IFX therapy also had increased levels of activated CD4+CD38+ T cells (C), CD4+ CD45RO+ T cells (D) and activated CD19+ CD38+ B cells (E) as compared to patients not initiating IFX therapy. (*p<0.05).

Patients with IBD had higher CD4+ T-cell percentages and lower levels of CD8+ T-cells in peripheral blood as compared to IBD negative controls, especially prior to IFX therapy (Figure 1A and 1B). Samples from the patients at the initiation of IFX therapy and patients on continuing IFX therapy had significantly lower levels of CD4+ T-cells, compared to patients not on therapy, approaching normal levels (Figure 1A). However, comparison of the no IFX therapy, and post-IFX as initial (one single infusion) and continuous (at least 3 dose regimen) therapy, data showed that the treatment led to a significant decrease in CD3+CD4+ (from ^≈^ 85% to 61% (initial), p=0.05 Figure 1A) and a significant increase in the mean percentage of CD3+CD8+ T-cells (from ^≈^ 10% to 29% and 26%, respectively, p=0.005, Figure 1B). The post IFX samples were obtained after IFX infusion. Following IFX administration for at least 3 months, the patients had a significant increase of CD4+CD38+ activated T-cell percentages (from ^≈^ 26% to 52%, p=0.03, Figure 1C). In addition, there was a significant increase in memory CD4+ T cell (CD4+CD45RO+) percentage not only during continuous treatment (from ^≈^ 24% to 47%, p=0.03, Figure 1D), but also immediately following the initial treatment (from ^≈^ 24% to 56%, p=0.04). There was a non-significant increase in activated B cells, CD19+CD38+ (from 18% to 42% and 45%, respectively, Figure 1E) during IFX therapy. 

### ANXA1 mRNA and protein levels are decreased in the peripheral blood of IBD patients

**Figure 2:**
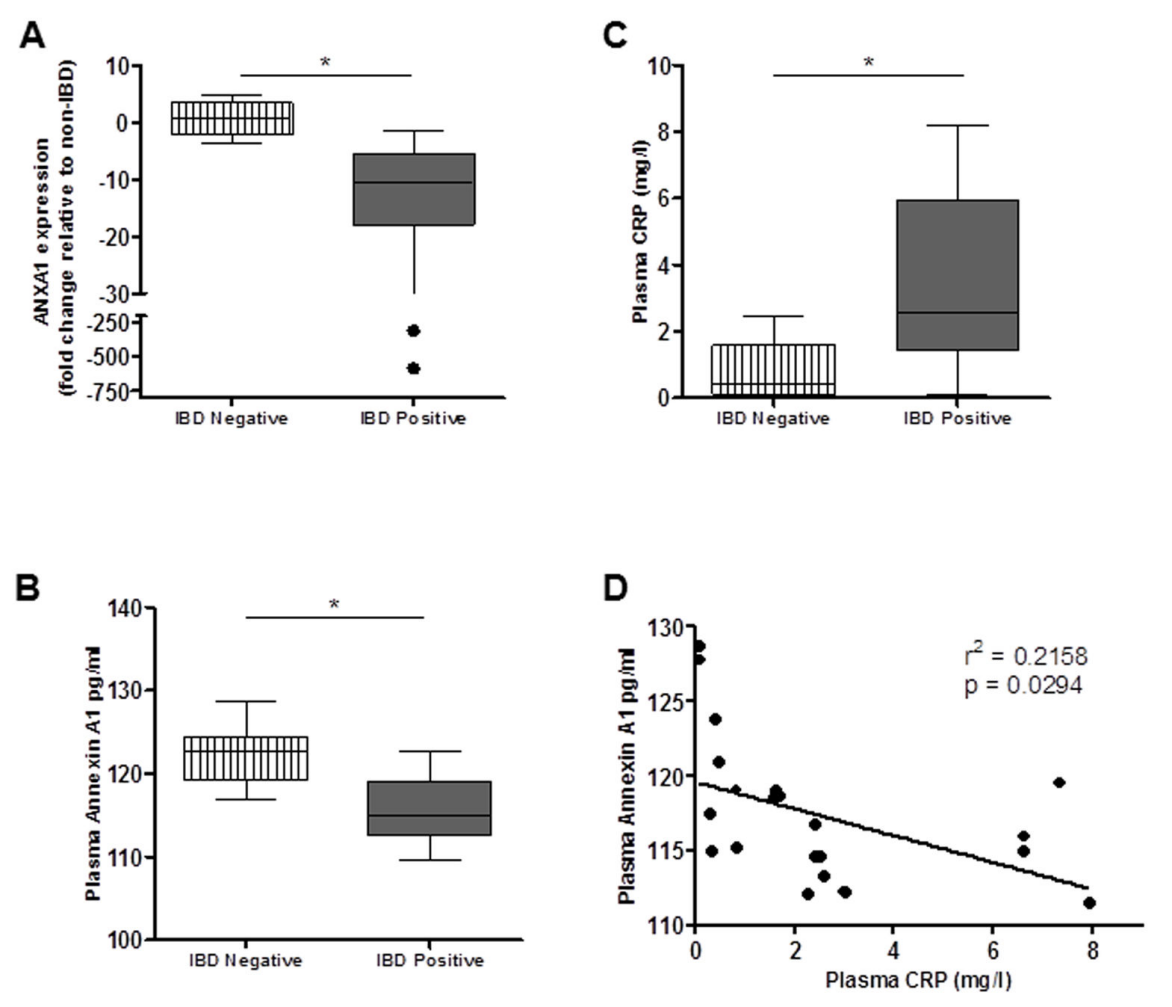
ANXA1 is expressed at lower levels in blood of IBD patients. (A) The level of ANXA1 mRNA was determined by real-time RT-PCR analysis from peripheral blood mononuclear cells was lower in IBD patients compared to healthy controls. (B) Plasma Annexin A1 levels were assayed using ELISA. Patients with IBD had lower levels of plasma ANXA1 compared to IBD negative controls. (C) Plasma CRP levels were measured by ELISA. Healthy controls had significantly lower levels of plasma CRP compared to patients with IBD. An inverse correlation was observed between plasma CRP and plasma ANXA1 (D). (*p < 0.05).

Measurement of ANXA1 gene expression in PBMC from patient groups showed that ANXA1 mRNA levels were significantly lower among IBD patients in comparison to healthy non-IBD controls (p<.05) (Figure 2A). The ANXA1 mRNA levels in PBMCs from IBD patients ranged from 2 to 500 fold lower than that of healthy controls. ANXA1 is generally attached to the plasma membrane and a fraction is shed in the plasma. We measured ANXA1 levels in plasma of IBD patients and healthy controls using ELISA. A previous study reported on decreased ANXA1 levels in plasma of patients with obesity [36]. We found a significantly lower level of free ANXA1 in plasma of IBD patients compared to healthy controls (unpaired T test, P<0.05) (Figure 2B). In contrast, a prominent marker of inflammation, plasma CRP, levels were significantly higher in patients with IBD compared to healthy controls (Figure 2C). A significant negative correlation was found between plasma CRP and plasma ANXA1 levels indicating that reduced levels of ANXA1 were associated with higher levels of inflammation (r^2^=0.2158; p=0.0294) (Figure 2D). 

### ANXA1 mRNA levels are reflective of the efficacy of IFX therapy

**Figure 3:**
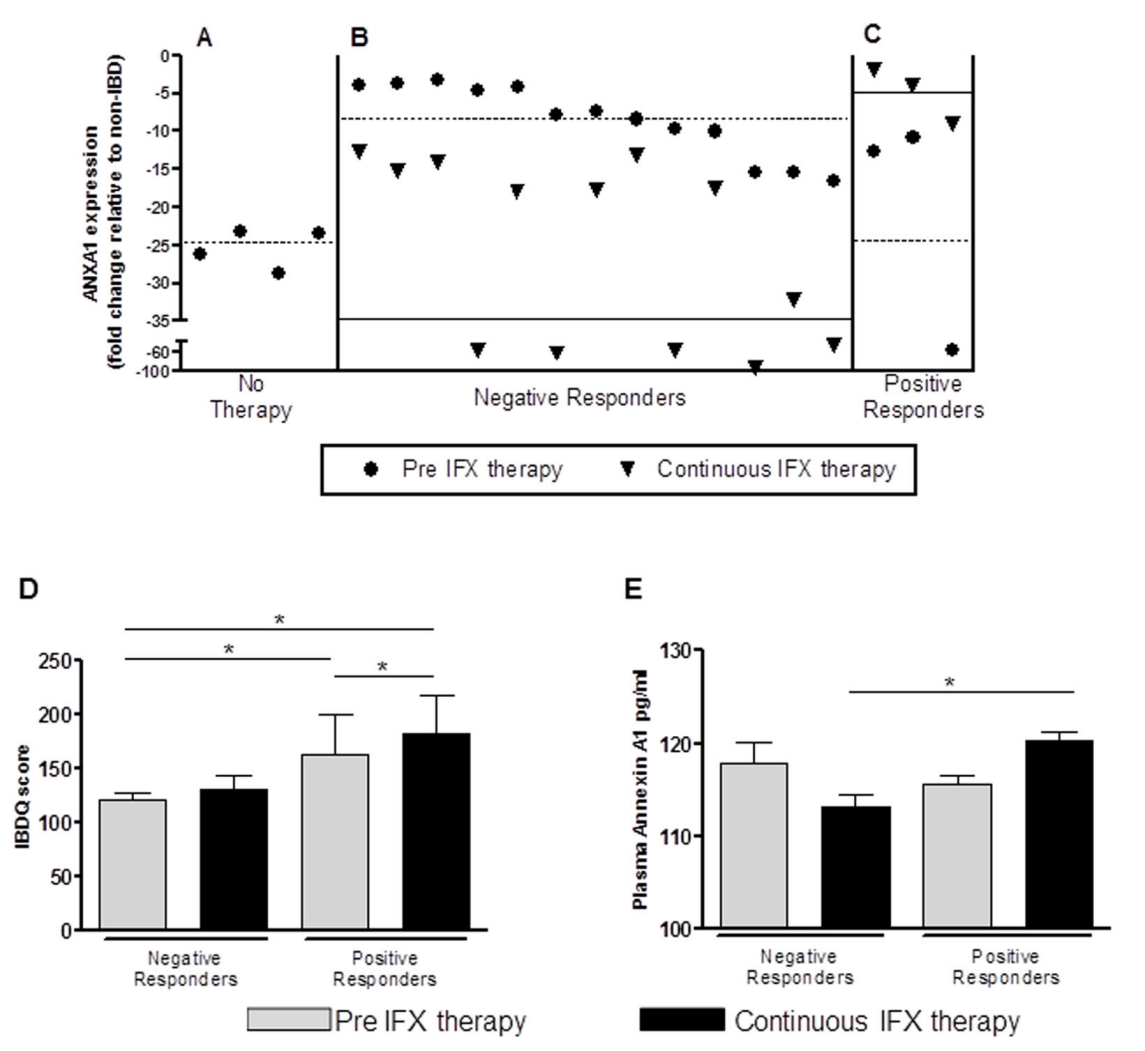
Analysis of longitudinal follow-up of IFX therapy influence on systemic ANXA1 expression. (A) IBD+ patients not on infliximab therapy; (B) IBD+ Patient group showing down-regulation of ANXA1 expression after continuous infliximab therapy; (C) Patient group showing up-regulation of ANXA1 expression after continuous infliximab therapy. The change in the transcriptional levels of ANXA1 between the 0 time point with no therapy and infliximab therapy showed 85% to be negative responders with a decrease in levels and 15% to be positive therapy responders with an increase in levels as compared to pre-therapy levels. The dashed lines are the mean of relative ANXA1 expression values at the first clinical attendance and the filled line are the mean ANXA1 levels following 3 months of infliximab treatment (longitudinal study of IBD patients n= 16). (D) Patients who were in the positive responder group had higher IBDQ scores as compared to those in the negative responder group both at baseline and following IFX therapy indicated a better clinical response in one group as compared to the other. Paired comparison of positive responders showed a significant increase in IBDQ score following continuous therapy. (E) Plasma Annexin levels were reflective of ANXA1 RNA levels in PBMCs. Similar trends were observed in the two patient groups as in PBMC. In positive responders there was an increase while in negative responders there was a decrease in plasma ANXA1 levels.(* p<.05).

Changes in ANXA1 expression during the course of IFX therapy are largely unknown. We determined the ANXA1 mRNA levels in PBMC of patients receiving IFX therapy. Analysis of longitudinal samples from 16 of the 28 IBD patients was performed. Four patients who were not on anti-TNF-α therapy and had no immediate plans to initiate IFX therapy were grouped as “no therapy” (Figure 3A). These patients had a lower level of ANXA1 transcripts in the PBMC compared to samples from patients initiating IFX therapy (Figure 3B, Table 2). Eighty-five percent of samples in the continuous therapy group clustered together in IFX/ANXA1 negative producers. This group had reduced ANXA1 expression in all patients at the time of the initiation of therapy (pre-IFX) compared to non-IBD healthy controls and sustained decrease through the subsequent monitoring (Figure 3B, Table 2). Fifteen percent of patients were termed IFX/ANXA1 positive producers, since a modest up-regulation of ANXA1 expression was observed over the course of IFX therapy in these patients (Figure 3C, Table 2). In this group, the post therapy ANXA1 levels approached that of healthy controls, the calibrator in this analysis (relative expression level of 1). Data collected from the IBD questionnaires from these participants further revealed that ANXA1 levels might have clinical significance to IBD. Negative responders were found to have a lower IBDQ score compared to positive responders either prior to therapy or following therapy (Figure 2D). A change of 16-32 in the IBDQ is indicative of a change in quality of life in patients with CD [37]. Following therapy all ANXA1 positive responders had a significantly higher IBDQ scores compared to pre therapy values (p<0.05) (Figure 2D). Plasma ANXA 1 levels also mirrored the finding in the PBMC (Figure 3E). Plasma ANXA1 levels were lower after long term therapy in negative responders while it increased over the course of therapy in positive responders. There was no correlation observed between other forms of immunosuppressive therapy (not IFX) and ANXA1 expression.

**Table 2:** Longitudinal follow-up of infliximab (IFX) treatment vs ANXA1 expression in IBD patients.

1-year longitudinal follow-up of patients	IFX (n=16)		No IFX (n=4)	p value^1^
ANXA1 expression	down-regulation (n=13)	up-regulation (n=3)		
first sample	-8.49 ± 1.32	-25.97 ± 14.33	-25.38 ± 1.27	0.004* ; 0.62
last sample	-34,95 ± 7.06	-4.92 ± 2.12		
p value^2^	0.0002*	0.10		

Note: n = total subjects.

Data are represented as mean ± SEM.

p values were obtained using Mann-Whitney test of comparison.

^1^ p value vs non-IFX group ^2^; p value vs first sample group.

* statistically significant differences.

### Increased TNF-α RNA expression is associated with IFX therapy

**Figure 4:**
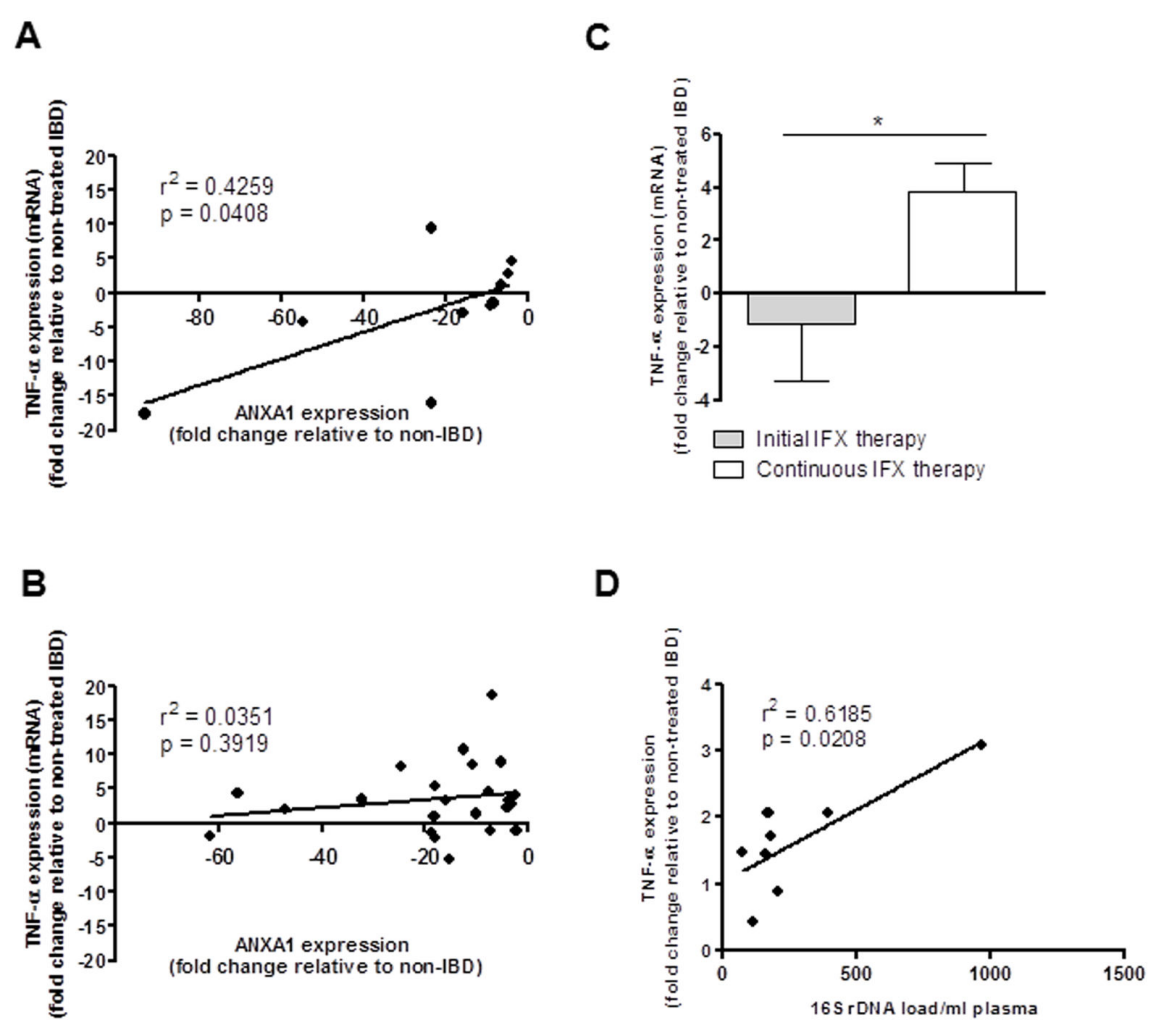
Correlation of TNF-α vs ANXA1 transcripts demonstrated an increase transcription of TNF-α following IFX therapy, also associated with plasma bacterial 16S load. The linear regression graphs (A) and (B) showed the relation between ANXA1 and TNF-α expression in the PBMC from patients prior to (A) and following IFX therapy (B), in IBD patients relative to IBD patients not initiating IFX therapy. (C) PBMC of IBD patients showed that following continuous IFX treatment there were higher transcriptional levels of TNF-α, compared to short term therapy, which could be due to compensatory mechanisms following ongoing TNF-α blocking therapy. (D) There was a significant positive correlation between TNF-α expression and increased bacterial load in patients with IBD (16S rDNA). (*p<0.05).

Correlation analysis demonstrated a significant positive association between ANXA1 and TNF-α transcripts in patients prior to the initiation of IFX therapy (Figure 4A). However comparisons of peripheral blood samples from initiation of therapy and continuous IFX therapy showed that the positive correlation was still maintained despite immunosuppressive therapy (Figure 4B). Measurement of TNF-α transcription showed that TNF-α mRNA levels were significantly increased in the PBMC from IBD patients following continuous IFX therapy compared to those receiving short term IFX (initial therapy) (Figure 4C) (p < 0.05). Interestingly, a significant positive correlation was observed between plasma 16S rDNA levels and TNF-α transcripts (r^2^ = 0.6185, p = 0.0208) (Figure 4D). No correlation was found between TNF-α mRNA expression and lymphocyte activation due to reduction of protein level by IFX. The increase in TNF-α transcription during IFX therapy may be a compensatory response to the neutralization of TNF-α protein.

### Bacterial 16S levels in plasma increases with IFX therapy and ANXA1 expression

**Figure 5:**
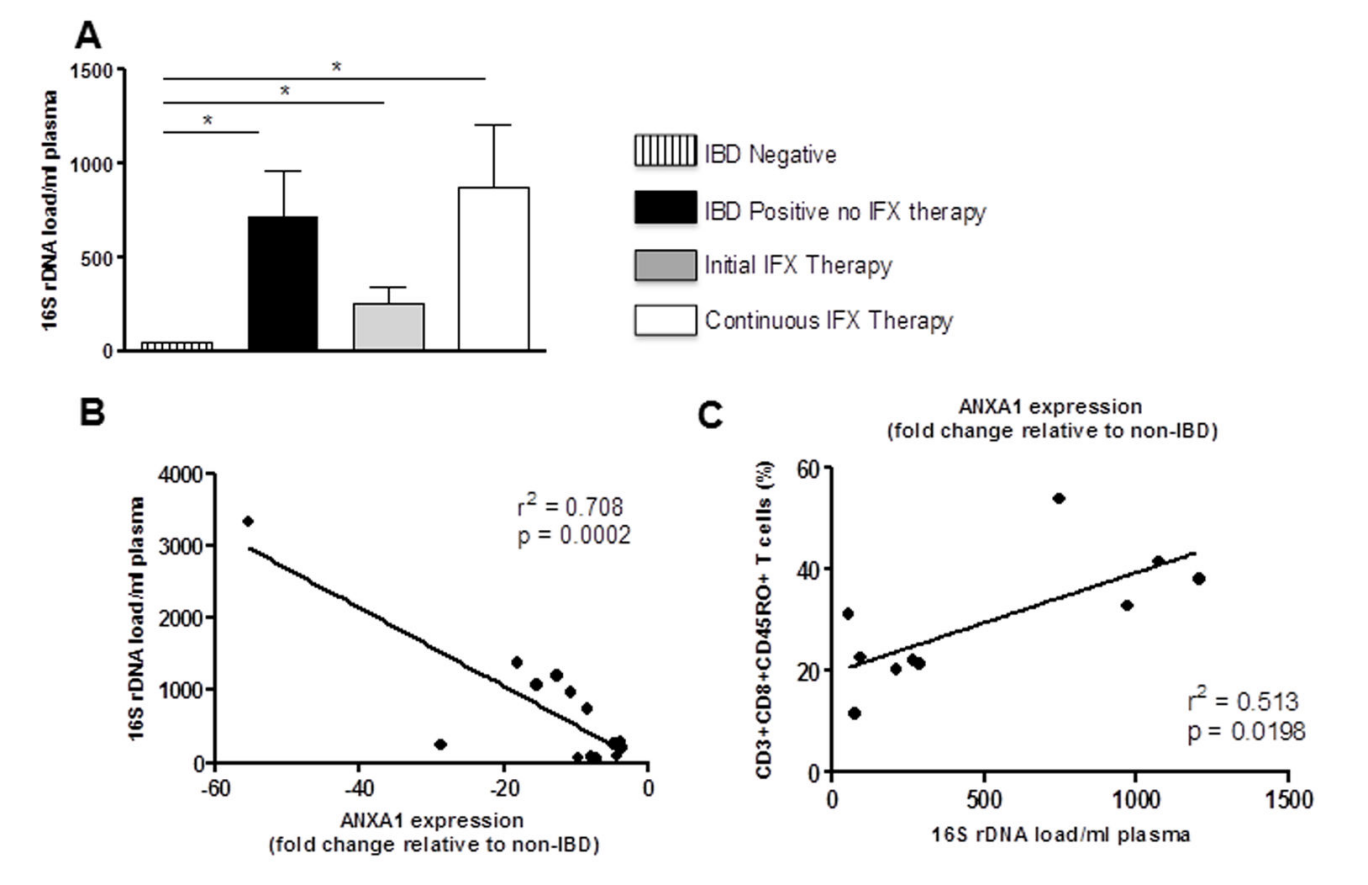
Plasma bacterial 16S ribosomal DNA (rDNA) correlates with IBD status, IFX therapy and ANXA1 expression. The number of 16S rDNA copies, was measured using quantitative PCR (qPCR). (A) An increase in the abundance of bacterial 16S rDNA in the plasma was observed in IBD patients (with and without IFX therapy) as compared to non-IBD controls. No significant difference was observed between patients initiating therapy and during IFX therapy. (*p<0.05) (B) An inverse correlation was observed between plasma 16S levels and ANXA1 expression. (C) A positive correlation was observed between plasma 16S levels and CD8+ CD45RO+ T cells (memory T cells).

Bacterial 16S rDNA levels were determined in plasma samples from IBD patients and healthy controls. Bacterial 16S levels in the plasma are reflective of mucosal barrier dysfunction and microbial translocation. Our data showed higher baseline levels of plasma 16s levels in patients with IBD as compared to healthy controls (Figure 5A). There was no significant difference of 16S levels in IBD patients on continuous IFX compared to patients not on IFX (p=0.07). A significantly lower level of ANXA1 expression is associated with a higher bacterial 16s DNA load in IBD patients (r^2^=0.708, p=0.0002) (Figure 5B). The impact of bacterial DNA load on T-cell activation was assessed by linear regression. There was a moderate positive correlation between memory CD8+ T-cell percentages and plasma 16S rDNA load (r^2^=.513, p=0.0198) (Figure 5C). 

### Decrease in ANXA1 expression in affected gut mucosa in Crohns Disease

**Figure 6:**
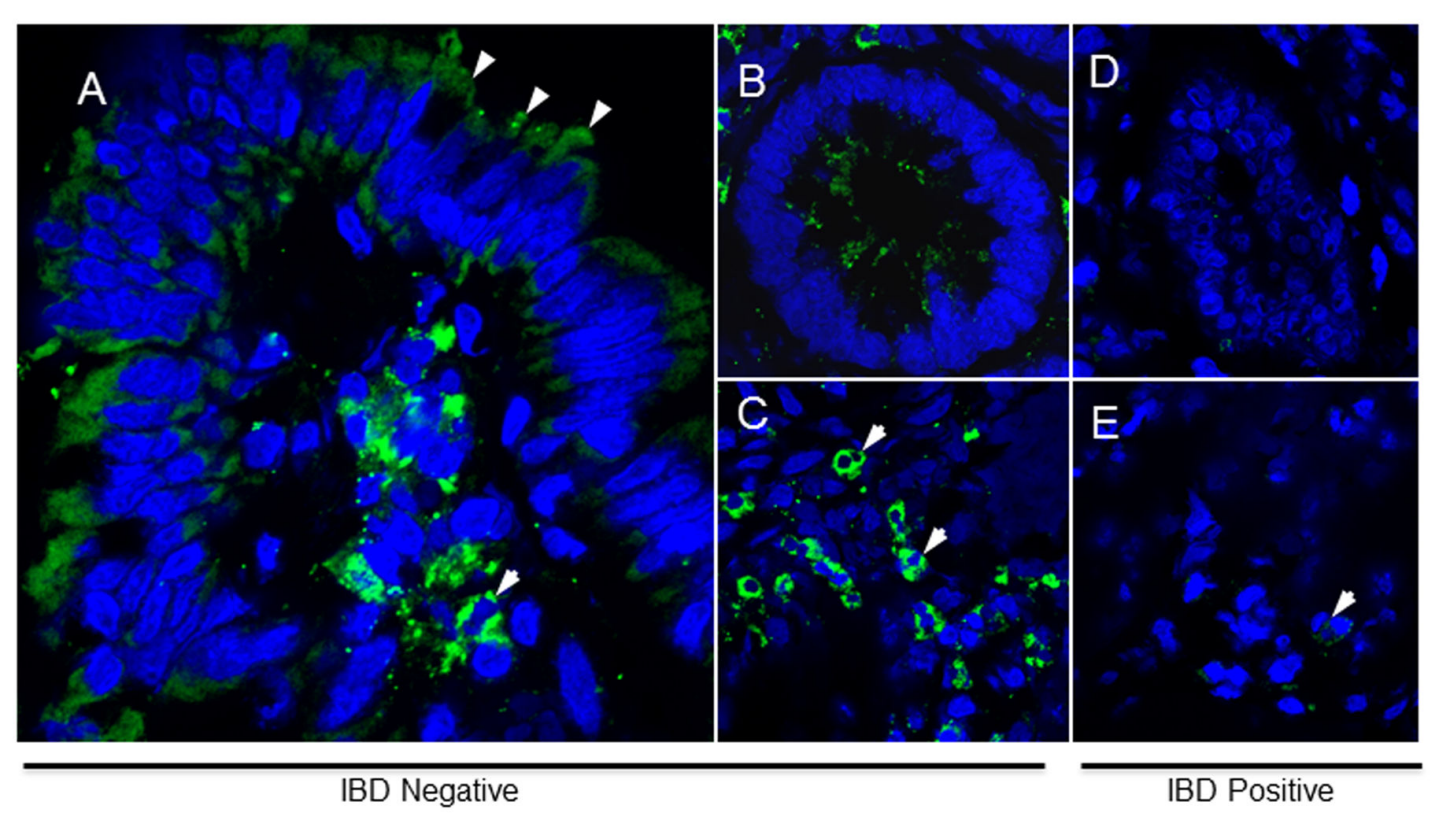
Decrease in ANXA1 levels in affected areas of the gut in patients with CD. Indirect immunofluorescence using a mouse anti-human ANXA1 antibody is shown with sections of colon resections from non-IBD (A-C) and IBD patients (D-E) by confocal microscopy analysis. (A) ANXA1 protein is localized in cytoplasm of the epithelial cell layer (arrowheads) and dispersed leukocytes in the mucosa in the crypt (B) and villus (C). (C) Non-IBD submucosal PMN (arrowheads) show increased levels of immunolabeling for ANXA1 in the cytoplasm in contrast to IBD patients. (D) ANXA1 protein is not detected in the crypt enterocytes and (E) weakly observed in some submucosal leukocytes (arrowhead) from IBD samples (Magnification 400×).

To examine the expression of ANXA1 transcripts and protein in the IBD affected colonic mucosa, we measured the colonic ANXA1 protein expression in inflamed colonic mucosa in CD and control tissues by fluorescent immunohistochemistry (Figure 6A-E). ANXA1 protein was readily detected in the cytoplasm and on cell surface of villus and crypt epithelial cells, and leukocytes dispersed in the connective tissue and lamina propria from normal mucosa (Figure 6A-C). In contrast, ANXA1 expression was significantly lower in the cells of IBD patients (Figure 6D-E). A remarkable loss in mucosal architecture was detected in the gut mucosa of patients affected by IBD and the findings were in agreement with previous studies [11].

## Discussion

 Pathophysiological mechanisms contributing to the development of inflammatory bowel disease have been intensely investigated, with a major focus on the inflammatory biomarkers. We report a substantial loss of ANXA1 expression in the colonic mucosal and peripheral blood compartments from patients with progressive IBD, specifically Crohns Disease. Our data suggest that ANXA1 transcript levels serve as an important biomarker of IBD [39]. Decreased ANXA1 levels in the colonic mucosa may support an increase in neutrophil recruitment, a common feature in IBD pathogenesis [30,40-42]. Increased intestinal tissue injury in the murine model of experimental colitis using ANXA1-/- mice [28] provides further support that ANXA1 is important for controlling intestinal inflammation and maintaining functional mucosa. Previous studies have reported ANXA1 imbalance in the blood of IBD patients, demonstrating that adults and children with CD or UC may have dysregulation in the ANXA1 associated immune signaling [43,44] and higher secretion of endogenous ANXA1 in the lumen from of UC patients [45]. 

Conventional treatment for IBD includes immunomodulators and biologic agents that reverse the immune dysregulation [38]. In this study, most of the IBD patients had moderate to severe inflammatory disease and were being treated with a combination of an immunomodulator (such as 6-Mercaptopurine or Methotrexate), and Infliximab to achieve disease remission. Longitudinal assessment of the peripheral blood samples was made at initiation of IFX therapy and at intervals during IFX therapy. The data demonstrates that systemic ANXA1 transcription levels are affected by initial and continuous IFX therapy. Despite the moderate recovery of ANXA1 transcripts at initial therapy, the majority of patients were IFX/ANXA1 negative producers, and continuous therapy may lead to a significant down-regulation of ANXA1 expression in the blood, resembling untreated patients. What is the significance of this downregulation of ANXA1 expression? ANXA1 is known to have multiple functions in modulating both innate and adaptive immune responses [26]. ANXA1 protein predominantly has an inhibitory effect on the innate immune response. The activation of ANXA1 can serve to inhibit cell trafficking, cytokine and superoxide radical release, subsequently inhibiting inflammation. In the adaptive immune system, ANXA1 promotes T-cell proliferation and activation [46]. It is possible that the downregulation of ANXA1 expression in our study may be due to the synergistic effect of both immunomodulating agents and infliximab on suppressing inflammatory immune response, thereby removing the trigger for underlying ANXA1 expression and mobilization. However, the confocal microscopic evaluation of colonic samples prior to the initiation of Infliximab therapy as well as following therapy showed that IBD patients had a substantial decrease or complete loss of ANXA1 protein expression in the colonic mucosa. This suggests that the lack of ANXA1 at the mRNA or protein level contributes to the pathogenic mechanisms underlying IBD. In addition, almost half of the patients lacking ANXA1 expression presented with a decreased IBDQ score. Interestingly, all IBD patients had lower levels of ANXA1 compared to heathy controls. This and other evidence suggests that ANXA1 auto-antibody levels were directly related to clinical disease in patients with Crohn's disease [43].

 Multiple mechanisms have been proposed for the efficacy of IFX in IBD patients. Besides the neutralization of TNF-α in its monomeric and trimeric forms, IFX causes apoptosis of activated T-cells and lamina propria lymphocytes by binding membrane bound TNF. Our findings showed that the level of TNF-α expression was increased in PBMC of IBD patients under continuous IFX therapy, suggesting a feedback regulation at the transcriptional level, a mechanism yet to be determined. The IBD patients were on immune modulating therapy, which is known to suppress proliferation of co-stimulated T-cells. With the inclusion of Infliximab, it is possible that patients may have decreased immune surveillance [47,48] and hence, bacteremia. Our data demonstrates a strong association between increased plasma bacterial 16S load and continuous combined therapy, with concomitant increase of TNF-α expression. The increase in bacterial 16S load suggests a baseline bacteremia present in patients receiving combined therapy. Whether detectable bacteremia has clinical relevance requires further investigation since these patients were clinically asymptomatic and did not display clinical evidence of septicemia. 

**Figure 7:**
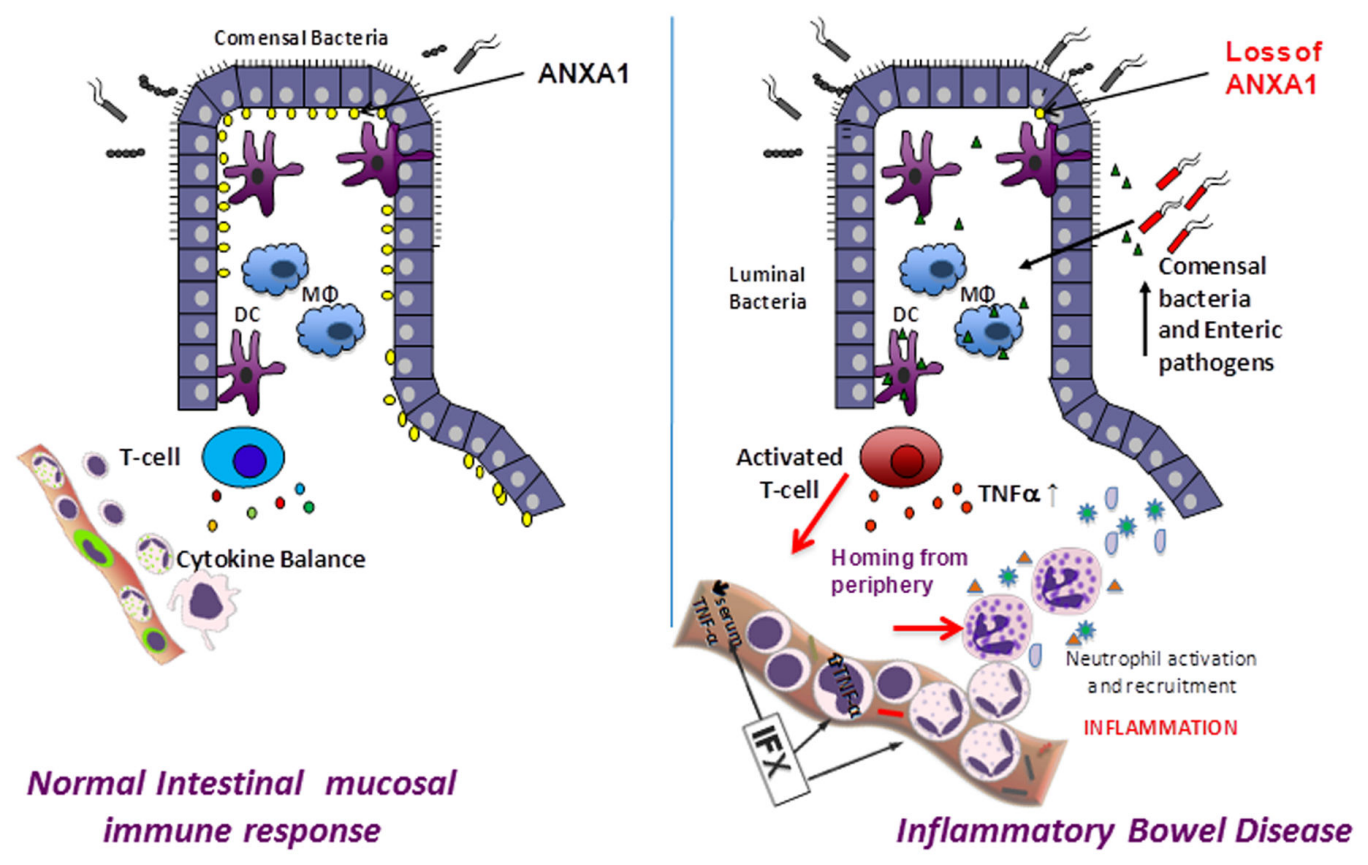
Proposed mechanism. The gut in an IBD patient is affected by the interplay of several factors including host microbe interactions, immune system defects/hyperactivity and dysfunctional epithelium. The loss or down regulation of Annexin A1 at the epithelial surface contributes to the epithelial dysfunction and immune modulation allowing for increased microbial translocation and increased inflammation despite IFX therapy. In positive regulators an increase in ANXA1 expression may improve control of inflammation with a reduction in symptoms.

 Our data demonstrates that lower levels of ANXA1 mRNA were associated with higher bacterial load in the plasma from IBD patients, and did not significantly correlate with TNF-α mRNA levels. Decreased ANXA1 in IBD patients as described here may induce an inadequate response to bacterial infection [49], phagocytosis signaling [50], and Th1-driven responses [46,51]. This chronic inflammation, observed in IBD patients, is further aggravated by a continuous stimulus of TNF-α by bacterial components in circulation. The disequilibrium between two signaling pathways, involving ANXA1 and TNF-α, may influence the disease pathogenesis via the loss of the immune homeostasis between two important pro-inflammatory and anti-inflammatory effectors. This may be a critical event for the chronic inflammation found in IBD patients, despite a successful therapy outcome at the initial states of treatment (Figure 7). 

 Activated T cells have been shown to cause gastrointestinal complications. We asked whether T-cell activation could be a contributing factor for the development of inflammatory condition in the gastrointestinal tract. Our results indicated that systemic T-cell activation correlated with increased bacterial load in plasma of IBD patients. Studies investigating the role of CD8 T-cells in bacterial infections have focused on cytokine secretion and cytotoxicity and if these cells also express CD45RO, a receptor correlated to activation or memory-type driven T-cells [52]. These cell types are activated to defend against bacteremia. This study demonstrates that continuous IFX therapy modified cellular immune activation in the blood from IBD patients, which was characterized by a significant disequilibrium in percentages of CD4+ and CD8+ T-cells, CD38+ T and B cells, and also memory CD4+ cells (CD45RO+). Similar results have demonstrated increased numbers of circulating CD8 T-cells in the peripheral blood of rheumatoid arthritis patients, observed three days after the IFX infusion. Those effects were attributed to the IFX therapy [53]. IFX possibly blocks homing of Th1 cells, thus cells temporarily accumulate in the peripheral blood. Our results from the initial samples and after continuous IFX administration, for at least 3 months, indicated the accumulation of not only memory and activated T-cells, but also activated B cells in the peripheral blood. 

 The ANXA1 levels in individual populations of T cells would give further insight into the role of individual T cells in the pathogenesis of IBD. However, our study design did not include analysis of purified cell populations from patient samples. Gender and ethnicity may also play a role in IBD disease progression. Although no significant associations were found in this study, the study was not powered to identify such differences. The present study provides clues to the complex inflammatory and anti-inflammatory mechanisms of IBD and warrants further analysis into the molecular basis of these pathways. We have identified for the first time a significant disequilibrium between ANXA1 and TNF-α expression in the blood of IBD patients. Future studies would include identifying the cellular mechanisms that reduce ANXA1 expression in IBD as well as a comprehensive clinical assessment including longitudinal IBDQ’s and CDAI’s. Furthermore, decreased ANXA1 expression in the colonic mucosa, increased TNF-α transcription, increased bacterial loads, and lymphocyte activation may pose challenges to the success of IBD therapy. Decreased ANXA1 expression may also partially explain the increased bacteremia and consequently the increased levels of TNF-α due to loss of anti-inflammatory action of ANXA1. ANXA1 as an important anti-inflammatory molecule may be a potential target for IBD treatment. Additional studies are needed to further define the role of ANXA1 in IBD.
